# Use of Oenological Tannins to Protect the Colour of Rosé Wine in a Bioprotection Strategy with *Metschnikowia pulcherrima*

**DOI:** 10.3390/foods12040735

**Published:** 2023-02-08

**Authors:** Maëlys Puyo, Scott Simonin, Géraldine Klein, Vanessa David-Vaizant, Natalia Quijada-Morín, Hervé Alexandre, Raphaëlle Tourdot-Maréchal

**Affiliations:** 1 UMR Procédés Alimentaires et Microbiologiques, Institut Agro Dijon, Université de Bourgogne Franche-Comté, Équipe Vin Alimentation Micro-Organismes Stress (VAlMiS), 21000 Dijon, France; 2 Changins, Viticulture and Enology, HES-SO University of Applied Sciences and Arts Western Switzerland, Route de Duillier 50, 1260 Nyon, Switzerland

**Keywords:** oenological tannins, bioprotection, colour, rosé wine

## Abstract

Although bioprotection is now recognised as an alternative to SO_2_ for limiting microbial spoilage, it does not guarantee protection against oxidation. This limits its application, more specifically for rosé winemaking. Oenological tannins present antioxidant properties, which could represent an interesting alternative to SO_2_ to protect must and wines against oxidation. A combination of the inoculation of a bioprotectant yeast strain and the addition of oenological tannins was tested to eliminate sulfites during the pre-fermentative step of rosé winemaking. In this experiment carried out in a winery, two oenological tannins were compared: quebracho and gall nut tannins. The antioxidant efficiency of tannins was compared to that of SO_2_. Colorimetric assays associated with chemical analyses of anthocyanins and phenolic compounds confirmed that the use of bioprotection alone did not protect the wine from oxidation. An addition of oenological tannins on musts stabilized the colour of bioprotected rosé wine in a similar way that SO_2_ addition did. Quebracho tannins appeared more efficient than gall nut tannins. The colour differences observed cannot be explained either by the concentration or forms of anthocyanins. However, the addition of tannins led to better protection of oxidation-sensitive phenolic compounds comparable to that obtained with the addition of sulfites.

## 1. Introduction

Reducing chemical inputs during winemaking has become a priority from a legal and societal point of view. The bioprotection of musts or grapes is a strategy to limit sulfiting during winemaking, and more specifically, during the pre-fermentative step. The bioprotectants currently proposed to winemakers are non-*Saccharomyces* yeast strains (most often belonging to the genus *Metschnikowia*) mainly used as pure cultures [1]. In both red and white winemaking, previous studies have demonstrated that bioprotectant strains colonise the matrix [2,3,4,5]. Their dominance suppresses or limits the development of indigenous yeasts, potential agents of wine spoilage [2,3,4], but remains unsatisfactory to ensure the protection of musts against oxidation.

The oxidation of a must is mainly an enzymatic phenomenon [6,7] due to tyrosinase, which belongs to the polyphenol oxidase family [7,8]. The presence of O_2_ in must also induces redox reactions through the interactions between phenolic compounds and oxygen, which can alter wine colour and taste through the production of off-flavours [9,10,11,12]. Oxidation of must compounds such as caftaric, coutaric, or hydroxy cinnamoyl tartaric acids generates the corresponding quinones. Those molecules can polymerise with each other and form brown pigments, producing enzymatic browning in wines [8,13]. Phenolic compounds can also be subjected to enzymatic oxido-reduction reactions mediated by Fe(III) and Cu(II) ions leading to production of quinones and hydrogen peroxide [14]. H_2_O_2_ is then used in the Fenton reaction, which oxidises wine’s compounds such as ethanol and phenolic compounds.

The addition of SO_2_ protects must and wine against enzymatic and chemical oxidation. It can interact with hydrogen peroxide and reduce it to water, thereby inhibiting the oxidation of other wine compounds [14,15,16,17]. SO_2_ can also minimise the production of quinones by converting them back into their corresponding phenols and then inhibiting quinone polymerization and subsequent wine browning [14].

To substitute the antioxidant and antioxidasic properties of SO_2_, the use of oenological tannin was investigated. Tannins are traditionally used to prevent protein haze and can promote colour stability [18]. Recently, the 2022 International code of good oenological practice of the International Organization of Vine and Wine recommended their use on must as an antioxidant [19]. Oenological tannins can be classified into two families: hydrolysable tannins and condensed tannins, also named proanthocyanidins. Hydrolysable tannins can be divided into two subfamilies: ellagitannins and gallotannins [20,21]. Ellagitannins are generally polymers of ellagic acid, gallic acid, and/or hexahydroxydiphenic acid such as castalgins or vescalgins and are mainly extracted from oak and chestnut wood [18,22]. Gallotannins are polymers of D-glucose and gallic acid. In most cases, they are obtained from gall nuts [18]. There are many different condensed tannins, which vary according to the monomeric units from which they are derived, their polymerisation degree, their galloylation level as a consequence of their botanical origin. They are derived from monomeric units of flavan-3-ols like catechin, epicatechin, epigallocatechin, or fistinidiol. Polymerisation of these compounds leads to the production of different condensed tannins such as profisetinidins (quebracho tannins), procyanidins (seed grape tannins), or a mixture of procyanidins and prodelphinidins (skin-grape tannins) [23].

The redox activity of gallotannins seems to be due to their ability to scavenge free radicals from the medium. Condensed tannins are able to scavenge peroxyl radicals, while ellagitannins chelate iron ions [24,25]. A previous study demonstrated that oenological tannins are able to inhibit polyphenol oxidases, such as laccase synthesised by the phytopathogen *Botrytis cinerea* [26]. This work demonstrated an antioxidasic efficiency of tannins on red and white wines, but with a larger effect on synthetic red wines than white ones. In addition, tannins can act as co-pigments by interacting with must anthocyanins [23,27] and can produce new pigments by direct or indirect condensation reactions with anthocyanins [28]. Because of the diversity of their properties, oenological tannins could represent an interesting alternative to replace or decrease the amount of SO_2_ used during winemaking and wine ageing.

To our knowledge, the antioxidant properties of tannins have not yet been studied in natural rosé wine, but only in white and red wines or in white wines supplemented with anthocyanins to simulate red or rosé wines [26]. Furthermore, tannins are not traditionally added during pre-fermentative steps in rosé winemaking. The present study, conducted in real conditions of production in a winery, aimed at assessing the efficiency of combining the antimicrobial properties of bioprotection with the antioxidasic/antioxidant properties of tannins (gall nuts or quebracho tannins) in comparison with bioprotection combined with SO_2_ addition at the pre-fermentative step during rosé elaboration. The implantation of bioprotection in musts and its effect on the development of alteration microorganisms during alcoholic fermentation were verified. The phenolic composition of wines and their colorimetric characteristics were determined to investigate the ability of oenological tannin addition to protect musts against oxidation and to stabilise the colour of bioprotected rosé wines.

## 2. Materials and Methods

### 2.1. Yeast Strains and Experimental Conditions

The bioprotectant strain *Metschnikowia pulcherrima* MCR 24 (Primaflora VB^®^) (AEB group—Kaysersberg, France). and the *Saccharomyces cerevisiae* strain Fermol Tropical^®^ (AEB group—Kaysersberg, France) were used. These strains were provided in dried form and were rehydrated before inoculation according to the manufacturer’s instructions. Fermentations were performed on must obtained from *Vitis vinifera* L. cv. Pinot Noir grape vines, grown in the vineyards of the University of Burgundy in Marsannay-La-Côte (Côte d’Or, France) and harvested in 2019. The vines were treated according to conventional viticulture conditions.

**Figure 1:**
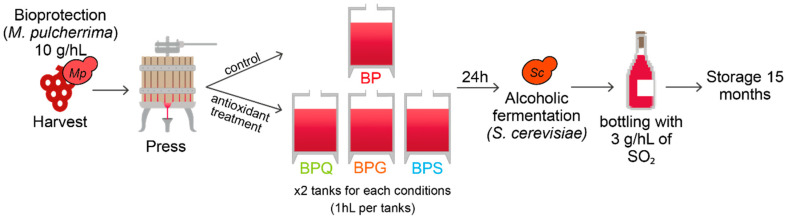
Shema of experimental conditions. BP: bioprotection (control modality), BPG: bioprotection and gall nut tannin addition (5 g/hL), BPQ: bioprotection and quebracho tannin addition (15 g/hL), BPS: bioprotection and SO_2_ addition (5 g/hL).

The grapevines were harvested by a mechanical grape harvester. The bioprotectant yeast was added in harvest containers at 10 g/hL (5 × 10^5^ CFU/mL) concentration, corresponding to the manufacturer’s recommendations. The juice obtained after pressing was distributed in eight 100 L flat-bottomed stainless steel tanks for alcoholic fermentation. Four modalities were designed (in duplicate): bioprotection (control modality named BP); bioprotection + addition of 5 g/hL of gall nuts tannin (GALLOVIN^®^) (named BPG); bioprotection + addition of 15 g/hL of quebracho tannin (PROTAN BIO Q^®^) (named BPQ); and bioprotection + addition of 5 g/hL of SO_2_ (named BPS) (Figure 1). Tannins were provided by AEB Group^®^ (Kaysersberg, France) and the concentrations used were those recommended by the manufacturer, based on the higher antioxidant capacity (three-fold) of gall nut tannins than quebracho tannins, in accordance with the literature [29].

Initial juice composition was 232 g/L of sugar, 493 mg/L of Yeast Assimilable Nitrogen (YAN) sources, with a total acidity of 6.83 g/L of tartaric acid and a pH value of 3.5. These values were obtained by Fourier transform infrared spectroscopy with OenoFoss© (FOSS—Nanterre, France) analyser.

At the end of alcoholic fermentation, wines were filtered through 0.22 µm filter cartridges with nitrogen gas. 30 mg/L of SO_2_ was added at bottling for each condition. Wines were stored in bottles with DIAM^®^ corks away from light for 15 months (samples named aged wines).

### 2.2. Experimental Sampling

Grape juice was collected directly from harvest containers before addition of the bioprotectant yeast (named T0 sample) and after pressing in the presence of the bioprotectant yeast (named T0 + BP). During alcoholic fermentation, samples were collected from both tanks for each condition: 24 h after vatting and before *S. cerevisiae* strain inoculation (named D1), 48 h after vatting (corresponding to 24 h after *S. cerevisiae* strain inoculation) (named D2), 72 h after vatting (named D3), 96 h after vatting (named D4), and at the end of alcoholic fermentation (D6).

### 2.3. Microbiological Analysis

Total yeasts counting was carried out on an agar plate medium WL Oxoid CM0309 with an addition of 0.2 g/L of chloramphenicol (Sigma-Aldrich, St. Louis, MO, USA). Yeast morphological diversity on WL medium allows discrimination between *Saccharomyces* genus, *M. pulcherrima* and *Hanseniaspora* genus [30] after an incubation of 48 h at 28 °C. On this medium, *M. pulcherrima* colonies appear red because of the production of a red pigment named pulcherrimin [31,32].

Enumerations of *Brettanomyces bruxellensis* were specifically made on agar plate ITV medium according to Gerbaux et al., [33] with some modifications (20 g/L glucose, 10 g/L yeast extract, 20 g/L tryptone, 0.1 g/L para-coumaric acid, 0.1 g/L ferulic acid, 0.03 g/L green bromocresol, 0.2 g/L chloramphenicol, 20 g/L agar, pH 5, with addition of cycloheximide 0.006% (*v*/*v*)) after an incubation of 7 days at 28 °C. Acetic acid bacteria (AAB) populations were determined by enumeration on Mannitol medium (25 g/L mannitol, 10 g/L yeast extract, 0.004% (*w*/*v*) Delvocid©, 0.002% (*w*/*v*) penicillin), and lactic acid bacteria (LAB) populations on Lac medium (78 mL grape juice, 33 g/L yeast extract, 0.6 mL/L Tween 80, 0.08 g/L MnSO_4_, 0.004% (*w*/*v*) Delvocid©, pH 5.1) after an incubation of 48 h at 28 °C.

### 2.4. Fermentation Kinetics and Wine Composition

Fermentation kinetics were monitored daily by densitometry. At the end of alcoholic fermentation (D6), each sample was analysed with Fourier transform infrared spectroscopy by OenoFoss^®^ for residual sugar (g/L), ethanol (% (*v*/*v*)), total acidity (g/L tartaric acid), and volatile acidity (g/L tartaric acid) concentrations of wines. SO_2_ analyses were realised on aged wines with a Sulfilyser+^®^ analyzer (Dujardin-Salleron, Noizay, France), according to the manufacturer’s instructions.

Combined SO_2_ (mg/L) can be calculated as the difference between total and free SO_2_ as follows:

$$\left[Combined\;SO_{2}\right]=\left[Total\;SO_{2}\right]-\left[Free\;SO_{2}\right]$$

### 2.5. Colorimetric Analyses by Tristimulus Coordinates (L*a*b*)

Colorimetric analysis of Tristimulus coordinates (L*a*b*) was performed on a spectrophotometer CM-5 Konica Minolta. The visible absorption spectrum was measured between 380 and 700 nm. The CIELab parameters (L*, a*, and b*) were obtained following the recommendations of the Commission Internationale de L’Eclairage (CIE, 2004) and the OIV-MA-AS2-11 method, using the standard illuminant D65 and the 10° standard observer on aged wines. Three millilitres of samples were transferred to a glass spectrophotometer cuvette (10 mm). The calculation of colorimetric parameters is done according to the OIV-MA-AS2-11 method.

Colorimetric variation between two samples ΔE* is calculated as follows:

$$\Delta E*=[{(\Delta L*)}^{2}+{\left(\Delta a*\right)}^{2}+{\left(\Delta b*{)}^{2}\right]}^{1/2}$$

Chroma of sample are calculated as follows:

$$C*=\surd \left({a*}^{2}+{b*}^{2}\right)$$

Hue of samples is calculated as follows:

$$h*=\arctan\left(b*÷a*\right)$$

### 2.6. Anthocyanins Analyses

For anthocyanins analyses, 500 mL of each wine was collected before bottling from each tank, and 0.2 g/L of sodium benzoate was added to the samples. Analyses of total anthocyanins by SO_2_ discoloration were carried out according to Ribéreau-Gayon & Stonestreet [34] methods. The concentration of total anthocyanins in mg/L was calculated as follows:

$$\left[Total\;anthocyanin\right]=({\mathrm{Abs}}_{\mathrm{Tube}\;A}-{\;\mathrm{Abs}}_{\mathrm{Tube}\;B}{)\;\times \;875}^{*}$$

The analysis of free and combined anthocyanins was carried out by PVPP column methods [35]. PVPP index (%) was calculated as follows:

$$PVPP\;index=\frac{\left[Total\;anthocyanins\right]-\left[Free\;anthocyanins\right]}{\left[Total\;anthocyanins\right]}\times 100$$

Combined anthocyanins concentration (mg/L) were calculated:

$$\left[Combined\;anthoycanins\right]=\left[Total\;anthocyanins\right]-\left[\;Free\;anthocyanins\right]$$

The ionisation index allows to determine the percentage of anthocyanins contributing to sample coloration [36]. Samples were centrifuged for 5 min at 9000× *g*. For each sample, Abs520 was measured in four different sample tubes by spectrophotometric analysis. The four sample tubes contained, respectively:

Tube 1 (Abs1): 5 mL of supernatant + 1 mL of distilled water

Tube 2 (Abs2): 5 mL of supernatant + 1 mL of NaHSO_3_ 15% (*v*/*v*)

Tube 3 (Abs3): 1 mL of supernatant + 7 mL of HCl 0.1 M + 2 mL of distilled water

Tube 4 (Abs4): 1 mL of supernatant + 7 mL of HCl 0.1 M + 2 mL NaHSO_3_ 15% (*v*/*v*)

Ionisation index (%) was calculated according to: $Ionisation\;index=\frac{\left({\mathrm{Abs}}_{1}-{\mathrm{Abs}}_{2}\right)\times 1.2}{\left(Abs-\mathrm{Abs}\right)\times \frac{100}{95}}\times 100$

### 2.7. UHPLC Analysis of Phenolic Compounds

Wine samples (bottles labeled as aged wines) were filtered on 0.45 µm Polytetrafluoroethylene (TPFE) filters and placed directly in UHPLC vials. The analyses were carried out in an Ultra High Performance Liquid Chromatography (UHPLC) system from Waters Acquity (Waters, Milford, MA, USA) with a Raptor ARC-18 (Restek) column (150 mm × 2.1 mm) with 2.7 µm of granulometry. The temperature of the column oven was regulated at 35 °C and the sample system was at 12 °C with an injection volume of 2 µL. Two eluants are used: a mix of water, 0.28% trifluoroacetic acid (TFA), and 5% methanol as eluant A, and methanol as eluant B, according to Popîrdă et al. [37].

The PDA (photodiode array) detector screens the UV-visible spectrum between 210 and 610 nm wavelength, with a 1.2 nm resolution and an acquisition frequency of 20 point/s. The fluorimeter records three pairs of excitation/emission wavelengths: 270 nm/322 nm, 270 nm/420 nm, and 330 nm/374 nm.

### 2.8. Statistical Analysis

The cellar condition of this experiment allowed two biological replicates for each condition. One-Way ANOVA test was performed for each analysis, followed by a Tukey test for group determination (α = 0.05). Statistical analyses and graphs were drawn with RStudio software (4.0.3 version).

Colorimetric similarity was analysed using multidimensional scaling (MDS). This multivariate statistical technique analyses the similarity relationships among samples, representing them as points on a map. The distances between pairs of points reflect the distances between the pairs of products. MDS was carried out on the symmetrical square distance matrix, where the rows and columns are the samples of the study, and ΔE* values are presented between each pair of samples.

## 3. Results

### 3.1. Microbial Behavour

Microbial populations were analysed in the grape juice collected directly from the harvest containers before bioprotection addition (T0) and at the end of pressing (T0 + BP). Before bioprotection addition, the indigenous yeast population in the must was approximately 4.8 × 10^6^ CFU/mL, and no *M. pulcherrima* was detected. Lactic and acetic acid bacteria populations were 1.0 × 10^3^ CFU/mL and 1.2 × 10^3^ CFU/mL, respectively. After bioprotection addition and grape pressing, the total yeast population reached 1.1 × 10^7^ CFU/mL with 70.5% (7.9 × 10^6^ CFU/mL) of *M. pulcherrima* yeasts.

The results of microbial analysis after juice transfer into tanks and the addition of tannins or sulfites are presented in Table 1. These analyses were performed at one (D1-before *S. cerevisiae* strain inoculation), two (D2-24 h after *S. cerevisiae* strain), and four days (mid-fermentation D4).

At D1, the *M. pulcherrima* population was approximately 3.1 × 10^6^ CFU/mL. This yeast was undetectable at D2 in BP and BPS conditions, whereas its concentration was maintained at 5 × 10^5^ CFU/mL in BPG and BPQ conditions. No *M. pulcherrima* colony was detectable at D4, whatever the condition (Table 1). Indigenous *S. cerevisiae* yeasts were detected at D1 in high concentration (around 6.4 × 10^7^ CFU/mL) without significant differences between conditions. At D2 (24 h after *S. cerevisiae* addition), *S. cerevisiae* concentration varied between 2.2 × 10^8^ and 2.8 × 10^8^ CFU/mL, and between 2.7 × 10^8^ and 3.3 × 10^8^ CFU/mL at D4. *B. bruxellensis* populations remained low (maximum of 3.50 × 10^2^ CFU/mL) at D1 and D2, whatever the modalities. From D4, no *B. bruxellensis* were detected. *Hanseniaspora* populations represented a small proportion of total yeasts (≈2% corresponding to 1 × 10^6^ CFU/mL) during D1 but they were undetectable from D2. Lactic acid bacteria populations decreased in all modalities to reach concentrations under 10^3^ CFU/mL at D4, except for the BPS condition (concentration of 1.48 × 10^3^ CFU/mL). Concerning acetic acid bacteria, the populations were below 1.0 × 10^3^ CFU/mL for BP and BPS conditions and 5.0 × 10^3^ CFU/mL for BPG and BPQ conditions, but those differences were not statistically significant (Table 1).

### 3.2. Fermentation Kinetics Analysis and Must and Wine Composition

Alcoholic fermentation ended in all tanks after 6 days (0.992 of density) (Table S1). At the end of fermentation, the residual sugar concentration was 1.71 (±0.26) g/L with a total acidity of 5.49 (±0.08) g/L of tartaric acid. The ethanol concentration was 13.71 (±0.07) % (*v*/*v*), and pH values were 3.25 (±0.03) (Table S1) (means values for the eight tanks). No significant differences were found in the evolution of density during alcoholic fermentation or in the final wine’s composition between the different modalities.

Concerning sulfite concentrations in wines after ageing (Table 2), the total SO_2_ concentrations were significantly different between conditions. BPS wines had the highest SO_2_ concentration (27 mg/L). BPG and BPQ wines presented lower levels at around 19 mg/L. The average concentration of free SO_2_ was 6 mg/L in all conditions, without statistical differences. Differences in total SO_2_ concentration could not be explained by the free SO_2_ form but by the combined SO_2_ form, where significant differences were found for BPS wines with the higher combined SO_2_ concentration (20.55 ± 2.83 mg/L).

### 3.3. Colorimetric Analysis by Tri-Dimensional Coordinates L*a*b*

**Figure 2:**
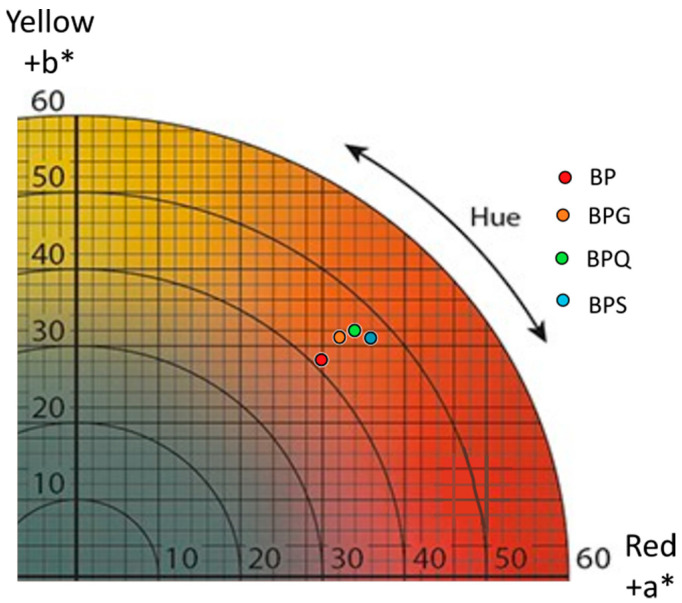
Graphic representing the CIELab coordinated a* (red hue) and b* (yellow hue) of the wines issued from four conditions. BP: bioprotection, BPG: bioprotection and gall nut tannin addition, BPQ: bioprotection and quebracho tannin addition, BPS: bioprotection and SO_2_ addition.

Parameters L*, a*, and b* correspond respectively to brightness parameters, red/green and yellow/blue hue of wine (Figure 2, Table S2a). BP wines had a higher L* value (L* = 67.56 ± 2.92) compared to those resulting from BPG (L* = 62.96 ± 0.81), BPQ (L* = 58.99 ± 1.92), or BPS conditions (L* = 60.61 ± 1.52), as well as lower a* and b* values than the wines that combined bioprotection and other treatments (Figure 2, Table S2a). The colorimetric variation (ΔE*) values, calculated with L*, a*, and b* parameters, were used to detect differences between samples. The ΔE* value is considered significantly different and distinguishable for the human eye (non-expert) at a threshold value of 3 [38]. The ΔE* values calculated for aged wines are presented in Table S2b. Comparisons between BPG/BPQ, BPG/BPS, and BPQ/BPS conditions showed ΔE* values up to the threshold value of 3 but close to it. Comparisons between BP and the three other conditions (BPQ, BPG, and BPS) show ΔE* values that are considerably higher than the threshold. The chroma (C*) and hue (h*) values (Table S2a) were also calculated from the a* and b* values. The hue (*p*-value = 0.10) and chroma (*p*-value = 0.0595) were not statistically different, but trends can be drawn for the chroma. BP wine had the lowest value (41.06 ± 3.46), BPG and BPQ wines had intermediate values, and BPS wine had the highest (47.70 ± 0.1).

Figure 2 shows the two-dimensional MDS space translating colorimetric differences between samples based on ΔE* computations. The stress value is very low, indicating a very good fit of the data.

**Figure 3:**
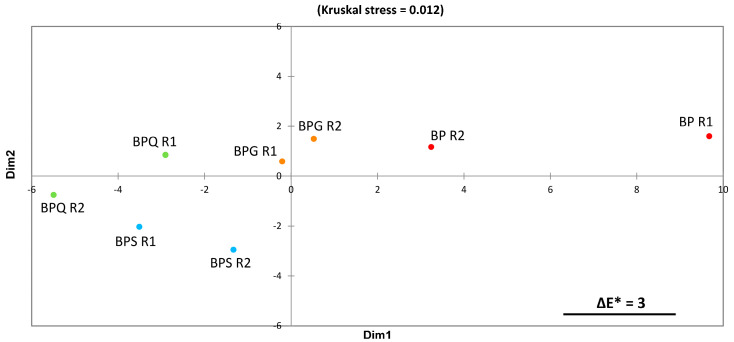
Colorimetric differences between samples obtained by MDS of the ΔE* distance matrix. The segment represented in the bottom right corner represents the distance corresponding to ΔE* of 3, which is the average human difference threshold for colour differences [38]. R1 and R2 correspond, respectively, to the two replicates of each condition. BP: bioprotection, BPG: bioprotection and gall nut tannin addition, BPQ: bioprotection and quebracho tannin addition, BPS: bioprotection and SO_2_ addition.

Figure 3 shows a clear visual proximity between the two BPS and the two BPQ replicates. BP is located on the opposite side of BPQ and BPS. BPG replicates are located between the two previous groups. Moreover, BP samples show low reproducibility, as visualised by the large distance between them.

### 3.4. Anthocyanin Analysis and Phenolic Compound Determination

#### 3.4.1. Anthocyanin Analysis by Spectrophotometric Analyses

After ageing, the mean total anthocyanin concentration in the wines was 17.2 (±2.24) mg/L (Table 3). Concentrations of free and combined anthocyanins were 2.92 (±1.90) mg/L and 14.20 (±1.95) mg/L, respectively. The ionization index allows determining the percentage of anthocyanins contributing to sample colouration. In wines, the ionisation index was about 37.9 (±20) %. No statistical differences were found between the four conditions.

#### 3.4.2. Phenolic Compound Analyses (UHPLC Analyses)

The concentration of mono-glycoside anthocyanins was 1.02 (±0.11) mg/L (Table 3). Other peaks were detected at 520 nm between the malvidin-3-glucoside peak and the end of the chromatograms, which corresponded to non-identified anthocyanin derivatives formed during winemaking and ageing. The same peaks at the same concentrations were detected for all the conditions.

The analysis of the aged wines revealed 14 phenolic compounds in addition to anthocyanins (Table 4). Significative differences between conditions were found only for gentisic acid (*p*-values ≈ 0.009), coumaric acid (*p*-values ≈ 0.037), and gallic acid (*p*-values ≈ 0.0002). Concerning gallic acid, a higher concentration (11.99 mg/L) was found in wines resulting from the BPG condition. Gentisic acid was detected at higher concentrations in wines resulting from the BPS and BPQ conditions (0.48 and 0.40 mg/L, respectively). For coumaric acid, the BP condition presented the lowest concentration (0.30 mg/L) and the BPQ condition the highest (0.38 mg/L), while the BPG and BPS conditions showed an intermediate concentration.

## 4. Discussion

### 4.1. Microbial Analyses

The bioprotective strain added to grapes in harvest containers was predominant and well established in the must after pressing (representing more than 70% of the total yeast population), despite the high concentration of indigenous yeasts in the must (4.8 × 10^6^ CFU/mL). This result confirms the implantation of the strain, as previously reported in the literature [2,3,4]. A decrease in the bioprotectant yeast was observed after 24 h. This drop in concentration could be explained by the quick start of fermentation.

*Brettanomyces* was detected in low concentrations and undetectable from D4, like the *Hanseniaspora* population, which was undetectable 24 h after vatting, regardless of conditions (Table 1). Bacterial populations (AAB and LAB) remained at low concentrations. Concentrations of undesirable microorganisms measured in this experiment were consistent with data previously observed in unaltered wines [39]. This lack of spoilage microorganisms in must and wine could be attributed to the implantation of the bioprotective yeast linked to the healthy state of grapevines.

### 4.2. Oenological Analysis of Wines

Our results indicated that the combined addition of *M. pulcherrima* and oenological tannins leads to a wine composition similar to that with SO_2_ (Table 2 and Table S1). Concerning sulfite concentrations in wines, the higher total SO_2_ concentration in the BPS condition can be explained by the addition of 50 mg/L in this condition in pre-fermentative steps. The free SO_2_ form was similar in all conditions, which suggests that the difference detected in total SO_2_ was due to a higher combined SO_2_ proportion in the BPS condition. These results could be attributed to higher acetaldehyde production by yeast. Studies have demonstrated that a higher initial SO_2_ concentration leads to increased acetaldehyde release by yeasts [40,41]. Acetaldehyde is the main compound that combines with SO_2_. However, the final concentrations appeared lower than expected. These results could be explained by the oxidation of SO_2_ and its transformation into other chemical forms (such as SO_4_^−^) during fermentation, and during ageing from the oxygen added during bottling [42].

### 4.3. Wine Colour

Wines resulting from the condition with tannins had higher a*, b*, and C* values, indicating higher colour intensity (Figure 2, Table S2a) and lower brightness (L* value) than the control BP wine (Table S2a), in accordance with the effect of tannins on wine colour previously observed in red wines and model wine solutions [43,44,45,46]. Both co-pigmentation [45] and the formation of anthocyanin-tannin adducts by direct or indirect condensation reactions could be responsible for the higher colour expression of the wines [22].

The visual proximity (Figure 3) between BPS and the conditions with tannins, and more specifically with BPQ conditions, suggested that these treatments gave quite similar colours, whereas the position of BP reveals a clear, perceptible difference in colour between BP on the one hand and the condition with antioxidant compounds on the other. These results are in accordance with the ΔE* values calculated (Table S2b) and the C* results. Moreover, BP samples showed low colour repeatability, which was already suggested by the high standard deviations on the L*, a*, b*, and C* parameters presented in Table S2a and the ΔE* values. These data underline that the use of bioprotection alone can impact rosé wine colour expression in an unpredictable way. On the other hand, the addition of oenological tannins allowed better colour reproducibility, as with SO_2_. Moreover, quebracho tannins induced a better colour expression than nut gall tannins at the doses studied. These first results obtained on rosé wines are in accordance with previous literature focused on white wine and the chemical properties of tannins [23,26].

### 4.4. Phenolic Composition of Wines

Spectrophotometric analyses showed similar concentrations of total anthocyanins in all conditions (Table 3). These results indicated that the total anthocyanin concentration in bioprotected wines was not impacted either by the addition of SO_2_ or oenological tannins. After storage, the main anthocyanins formed in wine were their combined forms in all treatments. In our experimental conditions, the addition of oenological tannins or SO_2_ did not impact the final combined anthocyanin concentrations (Table 3). The lack of differences for the ionisation index between conditions could be explained by the weak impact of the antioxidant compound tested on the proportion of anthocyanins, which contributed to wine colour, but the high standard deviation of the results did not allow further investigation into this result.

Concerning anthocyanin concentrations quantified by UHPLC methods, no significant difference was observed between wines. Only mono-glycoside anthocyanins were quantified, which are the only native anthocyanin form of Pinot Noir [47]. The concentration obtained (≈1 mg/L) corresponded to free anthocyanins and anthocyanins in weak co-pigmentation interaction with other wine compounds. This result could mean that oenological tannins, as well as SO_2_ addition, had no impact on the evolution of free or weakly bound anthocyanins. These concentrations were close to the concentrations of free anthocyanins obtained by spectrophotometric analyses. The colour differences observed in wines by CIELab analyses cannot therefore be explained by anthocyanin concentrations, by a different free/combined anthocyanin ratio, or by a coloured anthocyanin proportion.

Concerning the other phenolic compounds detected by the UHPLC method (Table 4), the higher gallic acid concentration in the BPG condition was explained by the addition of nut gall tannin in this treatment. It is a hydrolysable tannin composed of gallic acid units [22]. The gentisic and coumaric phenolic acids, which were in significantly different concentrations as a function of the conditions, are oxidation-sensitive wine compounds [48,49]. The concentration of these compounds was higher in the condition with antioxidant inputs (specifically SO_2_ or quebracho tannin) compared to the condition with bioprotection alone. This could suggest that the addition of an antioxidant compound (tannin or SO_2_) could protect certain oxidation-sensitive compounds during winemaking and ageing and explain their protective effect on wine colour.

## 5. Conclusions

The bioprotection of musts and grapes represents a strategy for limiting sulfite addition during winemaking. Although previous studies have demonstrated that bioprotectant non-*Saccharomyces* strains are able to protect musts and wines against microbial spoilage, they cannot protect must against oxidation. This fact can limit its practice, especially in rosé must, where it is crucial to preserve not only the freshness of aromatic grape varieties but also the colour after pressing.

This experiment carried out with the constraints of winemaking in the cellar led to a preliminary conclusion that the combined action of bioprotection and tannins could be an alternative to sulfites in pre-fermentative steps to guarantee microbiological protection and the colour of the wine, as shown by tristimulus coordinates and MDS analysis. In our experiments, it appeared preferable to favour quebracho tannins over gall nut tannins for better colour protection. The HPLC analysis showed that tannin addition protected oxidation-sensitive compounds (gentisic and coumaric acids) in a way comparable to the conditions with SO_2_. It will be interesting to carry out further experiments to study new pigments formed by interactions between anthocyanins, and between anthocyanins and other phenolic compounds to better understand the action of oenological tannins used as antioxidants in a bioprotected must.

This section is not mandatory but can be added to the manuscript if the discussion is unusually long or complex.

## Figures and Tables

**Table 1:** Microbial populations during winemaking.

Tank *	Day	Yeats (CFU/mL)	*Metschnikowia pulcherrima* (%)	*Saccharomyces* (%)	*Hanseniaspora* (%)	Lactic Acid Bacteria (CFU/mL)	Acetic Acid Bacteria (CFU/mL)
BP	1	3.2 ± 0.54 × 10^7^ **	2.45 ± 0.47	95.10 ± 0.93	2.45 ± 0.47	683 ± 24	1600 ± 471
BPG	1	7.6 ± 0.50 × 10^7^	7.15 ± 4.21	91.53 ± 4.13	1.33 ± 0.09	550 ± 24	1517 ± 212
BPQ	1	1.1 ± 1.3 × 10^8^	3.34 ± 2.67	95.64 ± 2.05	1.02 ± 0.62	567 ± 47	1500 ± 189
BPS	1	5.4 ± 5.3 × 10^7^	7.53 ± 0.23	91.31 ± 0.14	1.16 ± 0.09	567 ± 47	1267 ± 189
BP	2	2.8 ± 0.69 × 10^8^	ND ***	100 ± 0	ND	50 ± 24	100 ± 47
BPG	2	2.3 ± 0.02 × 10^8^	0.22 ± 0.31	99.78 ± 0.31	ND	ND	ND
BPQ	2	2.4 ± 0.02 × 10^8^	0.21 ± 0.30	99.79 ± 0.30	ND	83 ± 24	ND
BPS	2	2.2 ± 0.18 × 10^8^	ND	100 ± 0	ND	83 ± 24	ND
BP	4	2.9 ± 0.96 × 10^8^	ND	100 ± 0	ND	167 ± 47	817 ± 24
BPG	4	2.7 ± 0.16 × 10^8^	ND	100 ± 0	ND	600 ± 566	2133 ± 2923
BPQ	4	3.3 ± 0.15 × 10^8^	ND	100 ± 0	ND	417 ± 354	4733 ± 6505
BPS	4	3.2 ± 0.12 × 10^8^	ND	100 ± 0	ND	1383 ± 1815	300 ± 283

* BP: bioprotection, BPG: bioprotection and gall nut tannin addition, BPQ: bioprotection and quebracho tannin addition, BPS: bioprotection and SO_2_ addition. ** Results are the mean ± standard deviation of biological replicates, no significative differences are detected with the Anova test (α = 0.05). *** ND: not detected.

**Table 2:** SO_2_ composition of wines after ageing.

Condition *	Total SO_2_ (mg/L)	Bound SO_2_ (mg/L)	Free SO_2_ (mg/L)
BP	22.58 ± 0.86 ab **	15.58 ± 1.25 a	7.00 ± 0.59 a
BPG	18.64 ± 4.17 a	11.55 ± 4.05 a	6.20 ± 0.63 a
BPQ	19.79 ± 3.12 a	13.80 ± 3.17 a	6.03 ± 0.46 a
BPS	27.25 ± 2.42 b	20.55 ± 2.83 b	6.70 ± 0.97 a

* BP: bioprotection, BPG: bioprotection and gall nuts tannin addition, BPQ: bioprotection and quebracho tannins addition, BPS: bioprotection and SO_2_ addition. ** Results are the mean ± standard deviation of biological replicates, letter corresponds to statistical groups within each column, values with different letter are significantly different (Tukey test (α = 0.05)).

**Table 3:** Anthocyanins analyses by spectrophotometric and UHPLC methods.

	Spectrophotometric Analyses				UHPLC Analyses
	Total Anthocyanins (mg/L)	Free Anthocyanins (mg/L)	Combined Anthocyanins (mg/L)	Ionisation Index (%)	Anthocyanins (mg/L of Malvidine-3-O-glucoside)
BP *	16.19 ± 3.09 **	4.67 ± 0.82	11.52 ± 2.27	34.32 ± 22.88	1.07 ± 0.04
BPG	17.50 ± 3.71	2.63 ± 2.47	14.88 ± 6.16	47.95 ± 0.53	0.91 ± 0.08
BPQ	16.63 ± 0.00	2.19 ± 3.09	14.44 ± 3.09	48.62 ± 0.88	0.96 ± 0.12
BPS	18.38 ± 2.47	2.19 ± 0.62	16.19 ± 1.86	24.40 ± 34.51	1.16 ± 0.22

* BP: bioprotection, BPG: bioprotection and gall nut tannin addition, BPQ: bioprotection and quebracho tannin addition, BPS: bioprotection and SO_2_ addition. ** Results are the mean ± standard deviation of biological replicates, no significative differences are detected with Anova test (α = 0.05).

**Table 4:** Aged wines’ phenolic composition.

Compounds (mg/L)	BP *	BPG	BPQ	BPS
Caffeic acid	1.70 ± 0.08 a **	1.66 ± 0.02 a	1.66 ± 0.06 a	1.64 ± 0.12 a
Caftaric acid	63.54 ± 1.50 a	61.86 ± 1.93 a	63.11 ± 1.62 a	63.61 ± 1.21 a
Coumaric acid	0.30 ± 0.01 a	0.32 ± 0.03 ab	0.38 ± 0 b	0.35 ± 0 ab
Coutaric acid	11.20 ± 0.64 a	11.19 ± 0.42 a	11.74 ± 0.01 a	12.58 ± 0.57 a
Gallic acid	0.30 ± 0.01 b	11.99 ± 1.22 a	1.69 ± 0.73 b	0.33 ± 0.02 b
Gentisic acid	0.34 ± 0.01 a	0.34 ± 0 a	0.40 ± 0.02 ab	0.48 ± 0.04 b
Hydroxybenzoic acid	0.38 ± 0.07 a	0.41 ± 0.02 a	0.44 ± 0.05 a	0.51 ± 0.01 a
Protocatechuic acid	1.80 ± 0.37 a	2.01 ± 0.02 a	2.34 ± 0.12 a	1.83 ± 0.11 a
Catechin	1.44 ± 0 a	1.40 ± 0.04 a	1.36 ± 0.07 a	1.54 ± 0.19 a
B1 dimer	0.08 ± 0.01 a	0.07 ± 0.01 a	0.06 ± 0.01 a	0.07 ± 0.02 a
B2 dimer	0.02 ± 0.02 a	0.02 ± 0.01 a	0.02 ± 0 a	0.02 ± 0.01 a
Epicatechin	0.20 ± 0 a	0.18 ± 0 a	0.16 ± 0.01 a	0.11 ± 0.13 a
Grape Reaction Product	14.82 ± 0.81 a	14.74 ± 0.35 a	14.82 ± 0.1 a	14.70 ± 0.16 a
Hydroxytyrosol	0.39 ± 0.05 a	0.37 ± 0 a	0.39 ± 0.01 a	0.42 ± 0 a
Tyrosol	9.64 ± 0.26 a	9.56 ± 0.03 a	9.22 ± 0.25 a	8.96 ± 0.5 a

* BP: bioprotection, BPG: bioprotection and gall nut tannin addition, BPQ: bioprotection and quebracho tannin addition, BPS: bioprotection and SO_2_ addition. ** Results are the mean ± standard deviation of biological replicates, letter corresponds to statistical groups on each row, values with different letter are significantly different (Tukey test (α = 0.05)).

## Data Availability

Data is contained within the article or supplementary material.

## Supplementary Materials

The following supporting information can be downloaded at: https://www.mdpi.com/article/10.3390/foods12040735/s1. Table S1: Must composition at the end of alcoholic fermentation; Table S2: CIELab tristimulus coordinates and colorimetric variation (ΔE*) of aged wines.

## References

[1] Roudil L., Russo P., Berbegal C., Albertin W., Spano G., Capozzi V. (2020). Non-*Saccharomyces* Commercial Starter Cultures: Scientific Trends, Recent Patents and Innovation in the Wine Sector. Recent Pat. Food Nutr. Agric..

[2] Simonin S., Alexandre H., Nikolantonaki M., Coelho C., Tourdot-Maréchal R. (2018). Inoculation of *Torulaspora Delbrueckii* as a Bio-Protection Agent in Winemaking. Food Res. Int..

[3] Simonin S., Roullier-Gall C., Ballester J., Schmitt-Kopplin P., Quintanilla-Casas B., Vichi S., Peyron D., Alexandre H., Tourdot-Maréchal R. (2020). Bio-Protection As an Alternative to Sulphites: Impact on Chemical and Microbial Characteristics of Red Wines. Front. Microbiol..

[4] Windholtz S., Dutilh L., Lucas M., Maupeu J., Vallet-Courbin A., Farris L., Coulon J., Masneuf-Pomarède I. (2021). Population Dynamics and Yeast Diversity in Early Winemaking Stages without Sulfites Revealed by Three Complementary Approaches. Appl. Sci..

[5] Windholtz S., Vinsonneau E., Farris L., Thibon C., Masneuf-Pomarède I. (2021). Yeast and Filamentous Fungi Microbial Communities in Organic Red Grape Juice: Effect of Vintage, Maturity Stage, SO2, and Bioprotection. Front. Microbiol..

[6] Li H., Guo A., Wang H. (2008). Mechanisms of Oxidative Browning of Wine. Food Chem..

[7] Yang H., Tian T., Gu H., Li X., Cai G., Sun J., Wu D., Lu J. (2020). Analysis of Factors Related to Browning of Dangshan Pear (*Pyrus* Spp.) Wine. Food Chem..

[8] Oliveira C.M., Ferreira A.C.S., De Freitas V., Silva A.M.S. (2011). Oxidation Mechanisms Occurring in Wines. Food Res. Int..

[9] Coetzee C. (2014). Oxidation Treatments Affecting Sauvignon Blanc Wine Sensory and Chemical Composition. PhD Thesis.

[10] Hoenicke K., Simat T.J., Steinhart H., Christoph N., Geßner M., Köhler H.-J. (2002). ‘Untypical Aging off-Flavor’ in Wine: Formation of 2-Aminoacetophenone and Evaluation of Its Influencing Factors. Anal. Chim. Acta.

[11] Mayr C.M., Capone D.L., Pardon K.H., Black C.A., Pomeroy D., Francis I.L. (2015). Quantitative Analysis by GC-MS/MS of 18 Aroma Compounds Related to Oxidative Off-Flavor in Wines. J. Agric. Food Chem..

[12] Ugliano M. (2013). Oxygen Contribution to Wine Aroma Evolution during Bottle Aging. J. Agric. Food Chem..

[13] Simonin S. (2019). Etude de la Bio-Protection en œnologie.

[14] Danilewicz J.C., Seccombe J.T., Whelan J. (2008). Mechanism of Interaction of Polyphenols, Oxygen, and Sulfur Dioxide in Model Wine and Wine. Am. J. Enol. Vitic..

[15] Carrascón V., Bueno M., Fernandez-Zurbano P., Ferreira V. (2017). Oxygen and SO_2_ Consumption Rates in White and Rosé Wines: Relationship with and Effects on Wine Chemical Composition. J. Agric. Food Chem..

[16] Carrascón V., Vallverdú-Queralt A., Meudec E., Sommerer N., Fernandez-Zurbano P., Ferreira V. (2018). The Kinetics of Oxygen and SO_2_ Consumption by Red Wines. What Do They Tell about Oxidation Mechanisms and about Changes in Wine Composition?. Food Chem..

[17] Danilewicz J.C., Standing M.J. (2018). Reaction Mechanisms of Oxygen and Sulfite in Red Wine. Am. J. Enol. Vitic..

[18] Ugliano M., Slaghenaufi D., Picariello L., Olivieri G. (2020). Oxygen and SO_2_ Consumption of Different Enological Tannins in Relationship to Their Chemical and Electrochemical Characteristics. J. Agric. Food Chem..

[19] OIV Code International Des Pratiques OEnologiques. https://www.oiv.int/fr/standards/code-international-des-pratiques-oenologiques.

[20] Amarowicz R., Janiak M. (2018). Hydrolysable Tannins. Ref. Modul. Food Sci..

[21] Khanbabaee K., van Ree T. (2001). Tannins: Classification and Definition. Nat. Prod. Rep..

[22] Jourdes M., Pouységu L., Deffieux D., Teissedre P.-L., Quideau S., Ramawat K.G., Mérillon J.-M. (2013). Hydrolyzable Tannins: Gallotannins and Ellagitannins. Natural Products.

[23] Versari A., du Toit W., Parpinello G.P. (2013). Oenological Tannins: A Review: Oenological Tannins. Aust. J. Grape Wine Res..

[24] Magalhães L.M., Ramos I.I., Reis S., Segundo M.A. (2014). Antioxidant Profile of Commercial Oenological Tannins Determined by Multiple Chemical Assays. Aust. J. Grape Wine Res..

[25] Moilanen J., Karonen M., Tähtinen P., Jacquet R., Quideau S., Salminen J.-P. (2016). Biological Activity of Ellagitannins: Effects as Anti-Oxidants, pro-Oxidants and Metal Chelators. Phytochemistry.

[26] Vignault A., Pascual O., Jourdes M., Moine V., Fermaud M., Roudet J., Canals J.M., Teissedre P.-L., Zamora F. (2019). Impact of Enological Tannins on Laccase Activity. OENO One.

[27] Gombau J., Vignault A., Pascual O., Gómez-Alonso S., Gracía-Romero E., Hermosín I., Canals J.M., Teissedre P.-L., Zamora F. (2019). Influence of Oenological Tannins on Malvidin-3-O-Monoglucoside Copigmentation in a Model Wine Solution. OENO One.

[28] Remy S., Fulcrand H., Labarbe B., Cheynier V., Moutounet M. (2000). First Confirmation in Red Wine of Products Resulting from Direct Anthocyanin-Tannin Reactions. J. Sci. Food Agric..

[29] Vignault A., González-Centeno M.R., Pascual O., Gombau J., Jourdes M., Moine V., Iturmendi N., Canals J.M., Zamora F., Teissedre P.-L. (2018). Chemical Characterization, Antioxidant Properties and Oxygen Consumption Rate of 36 Commercial Oenological Tannins in a Model Wine Solution. Food Chem..

[30] Pallmann C.L., Brown J.A., Olineka T.L., Cocolin L., Mills D.A., Bisson L.F. (2001). Use of WL Medium to Profile Native Flora Fermentations. Am. J. Enol. Vitic..

[31] Kluyver A.J., van der Walt J.P., van Triet A.J. (1953). Pulcherrimin, The Pigment of *Candida pulcherrima*. Proc. Natl. Acad. Sci. USA.

[32] MacDonald J. (1965). Biosynthesis of Pulcherriminic Acid. Biochem. J..

[33] Gerbaux V., Briffox C., Dumont A., Krieger S. (2009). Influence of Inoculation with Malolactic Bacteria on Volatile Phenols in Wines. Am. J. Enol. Vitic..

[34] Ribéreau-Gayon P., Stonestreet E. (1965). Le Dosage Des Anthocyanes Dans Le Vin Rouge [Determination of Anthocyanins in Red Wine]. Bull Soc. Chim. Fr..

[35] Glories Y. (1978). Recherche Sur La Matière Colorante Des Vins Rouges. Ph.D. Thesis.

[36] Chris Somers T., Evans M.E. (1977). Spectral Evaluation of Young Red Wines: Anthocyanin Equilibria, Total Phenolics, Free and Molecular SO2, “Chemical Age”. J. Sci. Food Agric..

[37] Popîrdă A., Luchian C.E., Colibaba L.C., Focea E.C., Nicolas S., Noret L., Cioroiu I.B., Gougeon R., Cotea V.V. (2022). Carbon-Isotope Ratio (Δ13C) and Phenolic-Compounds Analysis in Authenticity Studies of Wines from Dealu Mare and Cotnari Regions (Romania). Agronomy.

[38] Martínez J.A., Melgosa M., Pérez M.M., Hita E., Negueruela A.I. (2001). Note. Visual and Instrumental Color Evaluation in Red Wines. Food Sci. Technol. Int..

[39] Guillamón J.M., Mas A., König H., Unden G., Fröhlich J. (2009). Acetic Acid Bacteria. Acetic Acid Bacteria.

[40] Park H., Hwang Y.-S. (2008). Genome-Wide Transcriptional Responses to Sulfite in Saccharomyces Cerevisiae. J. Microbiol..

[41] Jackowetz J.N., Dierschke S., Mira de Orduña R. (2011). Multifactorial Analysis of Acetaldehyde Kinetics during Alcoholic Fermentation by Saccharomyces Cerevisiae. Food Res. Int..

[42] Boulton R.B., Singleton V.L., Bisson L.F., Kunkee R.E. (1999). The Role of Sulfur Dioxide in Wine.

[43] Liu Y.-X., Liang N.-N., Wang J., Pan Q.-H., Duan C.-Q. (2013). Effect of the Prefermentative Addition of Five Enological Tannins on Anthocyanins and Color in Red Wines. J. Food Sci..

[44] Chen K., Escott C., Loira I., Del Fresno J., Morata A., Tesfaye W., Calderon F., Benito S., Suárez-Lepe J. (2016). The Effects of Pre-Fermentative Addition of Oenological Tannins on Wine Components and Sensorial Qualities of Red Wine. Molecules.

[45] Gombau J., Vignault A., Pascual O., Canals J.M., Teissedre P.-L., Zamora F. Influence of Supplementation with Different Oenological Tannins on Malvidin-3-Monoglucoside Copigmentation. Proceedings of the BIO Web of Conferences, 39th World Congress of Vine and Wine.

[46] García-Estévez I., Alcalde-Eon C., Puente V., Escribano-Bailón M. (2017). Enological Tannin Effect on Red Wine Color and Pigment Composition and Relevance of the Yeast Fermentation Products. Molecules.

[47] Zaffalon P.-L., Dienes-Nagy Á., Nardone D., Vuichard F., Koestel C., Rösti J. (2014). Anthocyanes libres des vins, une analyse pour différencier des cépages suisses. Rev. Suisse Vitic. Arboric. Hortic..

[48] Ribéreau-Gayon P., Dubourbieu D., Donèche B., Lonvaud A. (2006). Handbook of Enology, Volume 1: The Microbiology of Wines and Vinification.

[49] Waterhouse A.L., Sacks G.L., Jeffery D.W. (2016). Understanding Wine Chemistry.

