# Supplementary material for: Use of Oenological Tannins to Protect the Colour of Rosé Wine in a Bioprotection Strategy with Metschnikowia pulcherrima
Source: Foods. 2023 Feb 8;12(4):735. doi: 10.3390/foods12040735 (PMC9955556; doi:10.3390/foods12040735)
Supplement: Supplementary file 1 [file foods-12-00735-s001.zip › foods-2187601-supplementary.pdf]

Supplementary data

Supplementary data 1

Table S1. Must composition at the end of alcoholic fermentation.

Condition*	Total acidity (g/L tartaric acid)	Volatile acidity (g/L tartaric acid)	Ethanol (% v/v)	Sugar (g/L)	pH	Density
**BP**	5.53 ±0.03**	0.16 ±0.00	13.77 ±0.01	1.50 ±0.00	3.24 ±0.01	0.992 ±0.001
**BPG**	5.52 ±0.08	0.15 ±0.01	13.73 ±0.06	1.45 ±0.07	3.26 ±0.03	0.992 ±0
**BPQ**	5.49 ±0.12	0.15 ±0.01	13.70 ±0.06	1.90 ±0.14	3.26 ±0.05	0.992 ±0.002
**BPS**	5.44 ±0.10	0.13 ±0.02	13.64 ±0.11	2.00 ±0.00	3.27 ±0.02	0.992 ±0.001

*BP: bioprotection, BPG: bioprotection and gall nuts tannin addition, BPQ: bioprotection and quebracho tannins addition, BPS: bioprotection and SO_2 addition.

**Results are the mean ±standard deviation of biological replicates, no significative differences are detected with Anova test (α = 0.05)

Supplementary data 2

Table S2. CIELab tristimulus coordinates and colorimetric variation (ΔE*) of aged wines.

Table S2a. Tristimulus coordinates L* (brightness), a* (red hue), b* (yellow hue), the chroma (C*) and the hue (h* in degree) of aged wines.

Condition*	L*	a*	b*	C*	h*
**BP**	67.56 ±2.92**	30.32 ±1.92	27.67 ±3.04	42.32 ±3.46	42.32 ±1.34
**BPG**	62.95 ±0.81	32.55 ±0.28	31.36 ±0.40	45.20 ±0.08	43.93 ±0.62
**BPQ**	58.99 ±1.92	34.12 ±1.10	32.70 ±0.16	47.27 ±0.68	43.79 ±1.06
**BPS**	60.61 ±1.52	36.18 ±0.42	31.08 ±0.65	47.70 ±0.10	40.66 ±0.92

*BP: bioprotection, BPG: bioprotection and gall nuts tannin addition, BPQ: bioprotection and quebracho tannins addition, BPS: bioprotection and SO_2 addition.

**Results are the mean ±standard deviation of biological replicates,

Table S2b. Colorimetric variation (ΔE*) between conditions and replicates of aged wines

Comparison*	ΔE* **
BP rep 1 vs BP rep 2	6,55
BPG rep 1 vs BPG rep 2	1,34
BPQ rep 1 vs BPQ rep 2	3,14
BPS rep 1 vs BPS rep 2	2,41
BP vs BPG	6,30
BP vs BPQ	10,64
BP vs BPS	9,70
BPG vs BPQ	4,47
BPG vs BPS	4,33
BPQ vs BPS	3,08

[*]BP: bioprotection, BPG: bioprotection and gall nuts tannin addition, BPQ: bioprotection and quebracho tannins addition, BPS: bioprotection and SO_2 addition.

[**]Results are calculated with tristimulus coordinates table S2a.
